# An LSTM Network for Apnea and Hypopnea Episodes Detection in Respiratory Signals

**DOI:** 10.3390/s21175858

**Published:** 2021-08-31

**Authors:** Jakub Drzazga, Bogusław Cyganek

**Affiliations:** Department of Electronics, AGH University of Science and Technology, 30-059 Kraków, Poland; cyganek@agh.edu.pl

**Keywords:** sleep apnea, hypopnea, LSTM, deep learning, PSG

## Abstract

One of the most common sleep disorders is sleep apnea. It manifests itself by episodes of shallow breathing or pauses in breathing during the night. Diagnosis of this disease involves polysomnography examination, which is expensive. Alternatively, diagnostic doctors can be supported with recordings from the in-home polygraphy sensors. Furthermore, numerous attempts for providing an automated apnea episodes annotation algorithm have been made. Most of them, however, do not distinguish between apnea and hypopnea episodes. In this work, a novel solution for epoch-based annotation problem is presented. Utilizing an architecture based on the long short-term memory (LSTM) networks, the proposed model provides locations of sleep disordered breathing episodes and identifies them as either apnea or hypopnea. To achieve this, special pre- and postprocessing steps have been designed. The obtained labels can be then used for calculation of the respiratory event index (REI), which serves as a disease severity indicator. The input for the model consists of the oronasal airflow along with the thoracic and abdominal respiratory effort signals. Performance of the proposed architecture was verified on the SHHS-1 and PhysioNet Sleep databases, obtaining mean REI classification error of 9.24/10.52 with standard deviation of 11.61/7.92 (SHHS-1/PhysioNet). Normal breathing, hypopnea and apnea differentiation accuracy is assessed on both databases, resulting in the correctly classified samples percentage of 86.42%/84.35%, 49.30%/58.28% and 68.20%/69.50% for normal breathing, hypopnea and apnea classes, respectively. Overall accuracies are 80.66%/82.04%. Additionally, the effect of wake periods is investigated. The results show that the proposed model can be successfully used for both episode classification and REI estimation tasks.

## 1. Introduction

Sleep apnea is a common disorder, characterized by episodes of shallow breathing or cessation of breathing during sleep [1]. According to some studies as much as 23.4% of female and 49.7% of male adults can be affected [2]. Moreover, many cases remain undiagnosed [3]. Risk factors for sleep apnea include such aspects as obesity, low blood oxygen saturation and enlarged tonsils [4,5]. Results of untreated sleep apnea include daytime sleepiness, snoring, non-restorative sleep and concentration problems [6].

There are several standards specifying annotation of sleep disordered breathing events. By far the most commonly adopted are the rules proposed by the American Academy of Sleep Medicine (AASM) [1]. These guidelines describe prerequisites for annotating apnea and hypopnea episodes. Apnea is detected when there is at least 90% drop of breathing amplitude, and hypopnea occurs when the amplitude falls by at least 30% for ≥2 breaths. Additionally, an arousal or arterial oxygen desaturation drop not less than 3% must be present to score the episode as hypopnea [1]. Another standard for sleep disordered breathing scoring is the Sleep Heart Health Study (SHHS) method [7]. Both approaches emphasize the duration of an event. To be clinically important, an episode should last at least 10 s.

Apneas and hypopneas are further divided into obstructive and central categories [1]. Treatment of these two diseases can also be different [8]. In this work, however, the two types of events are not distinguished, and both obstructive and central episodes are classified together. This approach was chosen to limit the number of labels present in the dataset and focus strictly on apnea/hypopnea differentiation.

To streamline diagnosis, the apnea–hypopnea index (AHI) was introduced, that allows for disease severity assessment [6]. The AHI can be calculated as a total number of apneas and hypopneas lasting at least 10 s, divided by sleep duration expressed in hours. If no sleep staging data is available for an examination, the monitoring time can be used instead, producing respiratory event index (REI). REI can be a surrogate for AHI [1].

The golden standard in sleep disorder diagnosis is an attended polysomnography (PSG) examination. It involves recording of many channels, coming from various medical sensors. The most important for detection of sleep disordered breathing is the oronasal airflow signal. It can be recorded by a thermal sensor (either thermistor, thermocouple or polyvinylidene fluoride (PVDF)) or pressure transducer. The respiration effort signal allows for differentiation between types of episodes. Several technologies of sensors in form of belts are used for this purpose, namely respiratory inductance plethysmography, PVDF, piezoelectric, and pneumatic. Blood oxygen saturation is important for scoring of hypopnea events, and can be measured by standard pulse oximeter. Electrocardiography (ECG), electroencephalography (EEG), electromyography (EMG) and electrooculography (EOG) are also mandatory. The last biological signal that must be recorded is body position [1,9]. While very thorough, this method is also expensive. An examination takes place in a dedicated sleep laboratory and requires the constant supervision of a technician. Another option is a home sleep apnea diagnosis. This scenario implies the use of a much simpler device, called a polygraph, for recording a reduced set of channels. While not as complete as PSG, polygraphy has better availability and in most cases is sufficient for the sleep apnea diagnosis [10,11].

To further increase the number of tests that can be conducted and handled, automatic annotation algorithms were developed. The objective is to streamline the diagnosis by providing preliminary examination description for the medical personnel. Then, mistakes can be corrected, and there is no need to mark the evident episodes by hand. In the future, it could be possible that such an algorithm could be used alone, without further verification by a human [12].

Firstly, requirements for such an algorithm include good performance, as measured by typical metrics used in machine learning with strongly imbalanced datasets. Secondly, the architecture must also fulfill expectations of medical personnel. The episodes must be individually marked, and AHI calculated based on these markers. There are models available, that analyze the signal from the entire night and provide only an estimation of AHI [13,14]. While good for screening purposes, such an approach cannot provide initial labels of disordered breathing episodes for sleep technicians. Therefore, the aforementioned models are not useful for reducing the time spent by a human to annotate the recording. Nevertheless, AHI estimation remains an important functionality of the automatic sleep disordered breathing detection algorithm.

A reduced number of required channels would also be an advantage. There are algorithms that can operate on only one signal and provide locations of the sleep apnea episodes [15,16]. However, since this work aims for analyzing polygraphic examinations and not screening, channels commonly available in polygraphs can be used without further complication of the setup.

The last factor considered is division of the episodes into multiple classes. Most of the recent work focuses on detection of the disordered breathing episodes, and not on their classification [17]. However, according to the AASM rules [1], apneas and hypopneas should be scored separately. Such a distinction can improve quality of the diagnosis [18]. Existing approaches towards such classification often have limitations. In [19,20], 1D and 2D convolutional neural networks (CNN) have been analyzed. These works aim for providing separate labels for hypopnea and obstructive apnea events. Unfortunately, annotation is done on epochs of 30 s duration, and samples containing both hypopnea and obstructive apnea are discarded. Examinations without either of these classes were also removed from the study; therefore, performance of the algorithms for healthy patients is undetermined because of different breathing dynamics. This phenomenon of poor performance for less disordered cases was observed in [15]. In [21,22] performance of several deep learning methods in apnea-hypopnea distinction task is evaluated. For this purpose, a single-lead ECG signal is used. Limiting factors include lack of precise episode boundaries determination and exclusion of patients with central or mixed sleep apnea, as well as with cardiovascular diseases. Another interesting approach is documented in [16]: the SpO2 signal alone was used for detection and classification of apnea and hypopnea episodes separately. A window of 128 s with 50% overlap was chosen, thus temporal resolution of this approach is relatively low. With accuracy of hypopnea classification being only 22.78%, this method would require further improvement before being able to be used in a practical scenario. AHI estimation accuracy was also assessed, but unfortunately only for separating moderate and severe cases from normal and mild ones. Records with labeling flaws were discarded, as well as examination portions containing artifacts. Interesting approaches were presented by Koley and Dey [23] and Lee et al. [24]. They report good performance in apnea/hypopnea detection and classification task. Unfortunately, the databases used in these works do not seem to be publicly available; hence, the results were not compared with the proposed algorithm. There are methods of AHI estimation that were tested on the recordings from the SHHS-1 database that was used also in this work. Deviaene et al. [25] proposed a method based on random forest classifier processing features from the SpO2 signal. This algorithm is designed for binary classification, i.e., it does not separate apnea and hypopnea episodes. As stated in the paper, raw model output tends to be underestimated; hence, the linear regression afterwards was used to compensate this error. Olsen et al. [26] developed an algorithm that that takes the ECG signal as an input and provides locations of sleep disordered breathing episodes, but does not distinguish between them. This approach is based on bidirectional gated recurrent units and yields good results in AHI estimation. Additionally, Uddin et al. [27] presented a very interesting algorithm that is not based on deep learning architecture. Signal processing techniques are used instead, utilizing airflow and SpO_2_ signals. The accuracy of AHI classification is very good. Moreover, the architecture marks individual episodes and classifies them as apnea or hypopnea, but unfortunately no details about the accuracy of this differentiation were provided.

The presented work provides a unified machine learning architecture for the following two tasks. First, is an accurate annotation of sleep disordered breathing episodes with distinction on apnea and hypopnea ones. The resolution of the labels provided is high enough to enable use of the algorithm as an aid for sleep technicians. The second feature is good REI prediction capability. All of these functions are performed by the original architecture based on the long short-term memory neural network (LSTM). Our algorithm also introduces few techniques that can be used more generally in the field of sleep disordered breathing episode detection. Namely:

- Input signals phase shifting to align breaths from multiple sensors;
- Separate stage for detection of hypopnea beginning, i.e., the moment, where the breath amplitude actually drops;
- Episode thresholding using average labels score.

The approach that is close to this study with goals of apnea/hypopnea classification is described in [28]. In this work, the CNN was employed to deliver annotation with 1 s resolution. Multiple channels were then used to perform the analysis. Only periodic breathing and possible episodes were excluded from the study. However, our proposed method shows better results.

The rest of the paper is organized as follows. Descriptions of the used databases, and specification of the developed algorithm are covered in Section 2. Section 3 contains description of the conducted experiments and their outcomes. In Section 4 there are conclusions and discussion regarding the presented approach.

## 2. Method Description

### 2.1. Model Architecture

Proposed architecture utilizes the LSTM networks to find apnea and hypopnea episodes in the respiratory signals. The model operates on a subset of signals collected during standard at-home polygraphic recording. The required signals come from the following sensors:

- Oronasal airflow (thermocouple);
- Thoracic respiratory effort (RIP band);
- Abdominal respiratory effort (RIP band).

**Figure 1:**
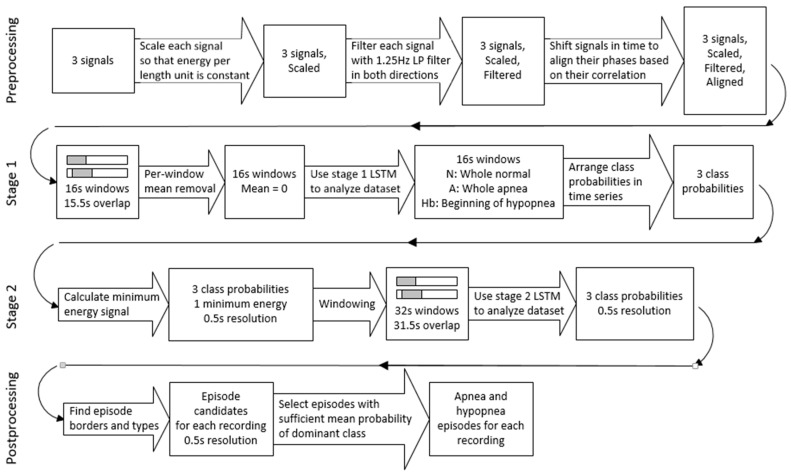
Block diagram of the proposed architecture.

An overview of our proposed architecture is presented in Figure 1. Details of each of its modules are described in the following sections.

LSTM is a type of recurrent neural network that has good capability of learning time dependencies in a signal. This and further properties of the LSTM networks are provided in the paper by Hochreiter and Schmidhuber [29].

To prevent overfitting, the designed system incorporates a dropout technique. It is a commonly used regularization method, which is described in [30].

In this work, apneas and hypopneas are treated as two different classes of disordered breathing. Obstructive, central and mixed apneas together form the apnea class, and obstructive, central and mixed hypopneas form the hypopnea class. The approach presented in this work focuses on utilizing raw respiratory signals. The model consists of preprocessing stage, two consecutive LSTM networks, and postprocessing stage. Two separate neural networks are used because of the way the architecture handles hypopnea episodes. First LSTM stage detects only the onset of hypopnea, and the second one determines the whole episode boundaries. Such an approach would not be possible with a single neural network. The stages of the model are discussed in detail in the following sections.

Input to the model consists of three respiratory signals: airflow collected from nasal cannula, thoracic respiratory effort and abdominal respiratory effort. These three time series are processed in parallel.

#### 2.1.1. Preprocessing Stage

In the preprocessing stage the input data for the first LSTM is prepared. Each signal’s offset is removed by subtracting mean value of the signal from each sample. The signals are then scaled to even out differences that occur between patients. To achieve this, a scaling factor (*SF*) for each signal is calculated. The signal is then multiplied by this value.

(1) $$SF=\sqrt{\frac{A\cdot N}{\sum_{n=1}^{N}{\left|x\left(n\right)\right|}^{2}}},$$

where *x* is the signal, *N* is the number of samples in the signal and *A* is a constant. The value of *A* was arbitrarily chosen for the signals after scaling to fall roughly within the (−1,1) range. The selected value was used for all signals through the experiment.

Additionally, airflow signal is inverted after scaling. Scaled signals are then filtered by the 8th order, 1.25 Hz low-pass Butterworth filter to remove noise. Filtering is done in both directions, so the original phase of each signal is maintained at this stage.

The last part of signal processing is phase alignment. It was observed in SHHS-1 database, that there are examinations where significant phase shift is present between the analyzed signals. In the Physionet database, however, there was no evident time lag between the used signals. Shifting two of the signals to match the third improved accuracy of classifying normal cases in the SHHS-1 database. Without airflow inversion and phase shifting 11% of normal cases were classified as normal, 59% as mild and 30% as moderate. No significant effect has been observed in the other AHI classes. Moreover, the technique slightly improved the performance of the model when trained on the SHHS-1 database and validated on the Physionet database. The increase in accuracy for the presented architecture was achieved only for apnea class. An average increase of 2.57% in correctly classified apnea samples was observed when running the same model on the same data with and without airflow inversion and phase alignment. Normal breathing and hypopnea results were not affected in our experiment. In order to achieve this phase shift, cross-correlation factors between signals are calculated and minimized by shifting two of the signals in time. The annotations are not shifted—they remain consistent with thoracic effort signal, which is not affected in this step. The final episode predictions also do not seem to be displaced—the presented improvement in accuracy was calculated with 0.5 s resolution; thus, it is sensitive to the exact episode locations.

**Figure 2:**
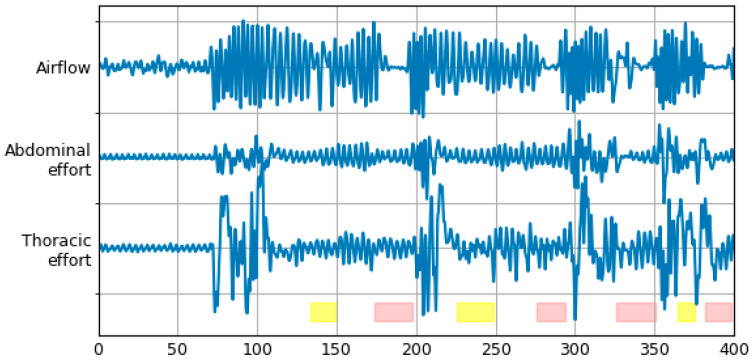
Respiratory signals from one of the SHHS-1 recordings, after preprocessing stage. At the bottom, there are bars representing labels from the database: red for apnea, yellow for hypopnea episodes.

An excerpt from one of the SHHS-1 recordings after preprocessing stage is shown in Figure 2.

#### 2.1.2. First LSTM Stage

After these initial steps, the signals are divided into windows. The length of each window is 16 s, and stride equals to 0.5 s. Hence, assuming 10 Hz sampling rate and three parallel signals, each sample size is 160 × 3. For each signal in every window, mean value was calculated and subtracted from each sample.

In this work, *a novel method of window annotation* for neural network training is proposed. In a typical approach each disordered breathing type has its own window class. These windows are then usually annotated based on either majority of window contents or on specific point in time (typically beginning, center or end of the window). However, in the presented method a different labeling scheme is proposed, which at first focuses on detection of the moment when hypopnea begins, and not on the whole episode itself. For the first LSTM, there are three classes of samples:

- Normal breathing (N);
- Apnea (A);
- Beginning of hypopnea (Hb).

In the case of normal breathing and apnea samples, the annotated class must be present in the whole window. However, the last class is different: the beginning of a hypopnea episode must be between 4 s and 12 s after the beginning of the window. This approach allows for more robust detection of hypopnea episodes. According to the AASM rules, hypopnea is annotated when at least 30% reduction in breathing amplitude occurs [1]. This amplitude drop can only be observed on the beginning of the episode. After that, breathing during hypopnea episode can closely resemble normal breathing patterns, thus being hard to discern. The amplitude of normal breathing can also change between patients, or even during the night, making detection of hypopnea onset a must. Windows that do not fall in any of these categories are discarded during training. The dataset is then balanced by removing excess windows of two major classes, to equalize number of samples with the least represented class (usually apnea). Before that, samples are randomized so each window has equal chance of getting to the final dataset.

Structure of the proposed neural network is as follows:

- LSTM layer—150 units;
- Dropout—factor 0.5;
- Dense layer—30 units, ReLu activation function;
- Dropout—factor 0.5;
- Softmax layer—3 units.

**Figure 3:**
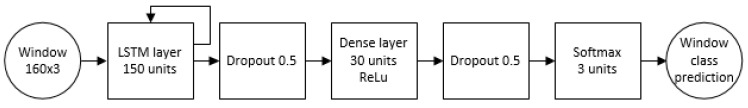
Signal propagation through the LSTM architecture.

Information flow is illustrated in Figure 3.

Such a model is trained with the Adam optimizer [31] for 50 epochs.

**Figure 4:**
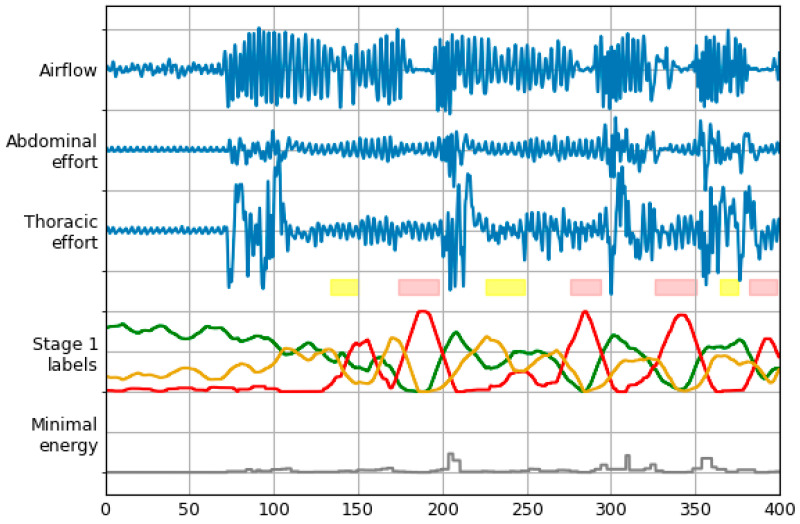
Signals and labels from Figure 2 with labels timeline produced by Stage 1 LSTM. Normal, apnea and hypopnea beginning classes are represented with green, red and yellow lines, respectively. Grey line represents the fourth signal that is input to Stage 2 LSTM.

This network is used to construct time series of N/A/Hb probabilities for each examination. To achieve this, class probabilities for consecutive windows are lined up. Temporal resolution of these time series is equal to window stride from the previous stage (0.5 s). A moving average filter is then applied, with window size of 32 samples (16 s). In this way, every sample reflects a mean probability of every window it was included in Figure 4 represents the same signals as presented in Figure 2, along with labels constructed by the first LSTM stage for that excerpt. One of the models from the 5-fold cross-validation experiment was used to obtain these data. The analyzed recording was not present in the training dataset of this model.

#### 2.1.3. Second LSTM Stage

The three constructed probabilities are used as an input to the next neural network. Additionally, from the preprocessed respiratory recordings fourth signal is calculated. It is needed to facilitate detection of the hypopnea end stage, since it is not detected by the first neural network and no other input to the second LSTM contains this information. The procedure for obtaining this signal is described below.

Zero crossings are detected in the airflow signal. Points obtained are then used to partition the recording into single inspirations and expirations. For each segment in every signal, energy *E* is calculated and scaled according to the following equation:

(2) $$E=\frac{\sum_{n=1}^{N}{\left|x\left(n\right)\right|}^{2}}{N^{2}},$$

where *x* is the signal, and *N* stands for the number of samples in the analyzed part of the signal. The desired time series is then constructed by filling spaces between consecutive zero crossings using the lowest from the three values calculated for this period. This signal is then downsampled to 2 Hz by decimation to match the rest of the time series prepared in this stage. At last, this time series is normalized by dividing each sample by the signal’s maximum value. This signal is illustrated in Figure 4 as “Minimal energy”. The addition of such data to the second LSTM resulted in increased accuracy of the classification.

The four signals of this stage are divided into widows. For second neural network, window length is set to 32 s with 0.5 s stride. With temporal resolution of 0.5 s this division results in samples of size 64 × 4. Annotation of these samples is done differently than for the first neural network. As this stage needs to output probabilities for two disordered breathing classes (apnea and hypopnea) plus normal breathing class, suitable labels have been introduced (N, A and H). Each sample’s class is determined based on the ending point of the sample. This approach is similar to the one presented in [15], but with addition of the third class. The dataset is balanced using the same method as before, i.e., randomization and removing samples from overrepresented classes. Each class has equal number of samples in the final training dataset.

**Figure 5:**
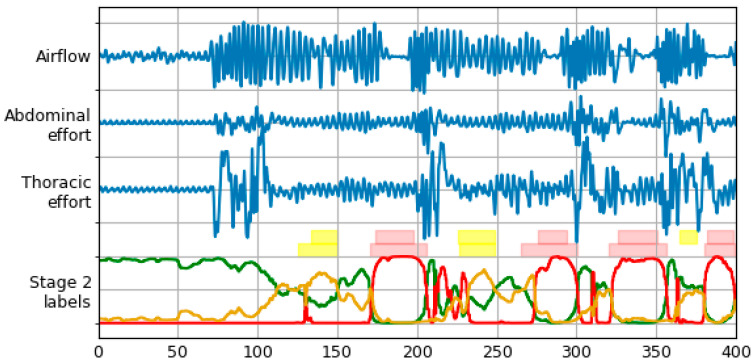
Signals and labels from Figure 2 with labels timeline produced by Stage 2 LSTM. Normal, apnea and hypopnea classes are represented with green, red and yellow lines, respectively. Two rows of label bars are present—top row for database (true) labels, and bottom row for final labels predicted by the model (after the postprocessing stage).

The architecture of the neural network used for final prediction is the same as in the previous stage. Training is done for 100 epochs using the Adam optimizer. Figure 5 illustrates an effect of operation of the proposed method.

#### 2.1.4. Postprocessing

The postprocessing stage is the last part of the algorithm. Its goal is to determine final apnea and hypopnea episode locations using window labels produced by the second neural network. The input to this stage is made by stacking class probabilities produced by the previous stage for consecutive windows. The resulting time series has temporal resolution the same as before, which is 0.5 s.

Transformation begins with finding labels with maximum probabilities for each point in time. Then, the borders of the episodes are annotated where the label with maximum probability changes. If the change is from N to A or H, start of an episode is marked. Alternatively, transition form A or H to N means an end of an episode. Shifts between A and H labels are not taken into account here.

The next step is to determine class affiliation of each episode. The assignment is done to the class, where majority of windows in given episode have maximum probability. Certainty of each episode is then calculated as arithmetic mean of these probabilities.

Obtained episodes are thresholded to filter out ones with low certainty. Threshold values used in this step are separate for apnea and hypopnea episodes. Their values are obtained by calculating AI and HI indexes for several different thresholds on a subset of examinations from the training set that were not used in neural network training process. Final thresholds are selected by minimizing the root-mean-square error (RMSE) of AI and HI indexes. Episodes with duration lower than 10 s are excluded from threshold determination, as well as from final AI and HI calculation.

Additionally, the episodes with low mean probability of N class, that were not previously marked, are annotated. To calculate the N threshold used for selection of these episodes, the higher from apnea and hypopnea thresholds is subtracted from one. All episodes with N probability below this threshold are marked as either apnea or hypopnea, depending on the class of majority of windows in such episode. This additional step is needed because there are episodes for which the model does not clearly show their class. In such episode, A and H probabilities are similar, and N probability is very low. It is clear that this is indeed an episode, but its type is hard to determine.

An effect of postprocessing stage transformations is shown in Figure 5. The bottom row of label bars was constructed using procedure described above. Merging of the two episode types with final class determination can be observed, as well as performance of different threshold levels for both classes.

This method of episode detection and filtering allows the model to be more accurate on unseen data. This is due to the fact that individual episodes are not fragmented when the threshold rises.

### 2.2. Implementation Details

The neural networks used in the proposed model have been implemented in Keras [32] with TensorFlow [33] backend. All experiments described in this paper were run on a standard PC. Images and graphs were prepared using Matplotlib [34].

### 2.3. Signals and Databases

In this study the SHHS-1 database was used [7,35]. Out of its 5804 records, 3610 were automatically selected based on the signal energy of the respiratory signals. For each of the three signals used, their energy has been calculated and divided by the length of the examination. Records having at least one signal with energy below specified threshold were excluded from the experiment. The threshold was arbitrarily chosen to filter out records with large parts indistinguishable from noise. From the 3610 remaining examinations, the first 1000 were used for validation of the proposed method. This truncation was done solely to limit computation time on available hardware. No additional record selection processes were performed. The advantage of this approach is that it can be reproduced during inference on new data—the energy of each signal can be calculated to indicate whether the record is of quality high enough to expect good results. The final collection contained recordings of 486 female and 514 male patients. Among them, 1.7% were healthy, 13.4% had mild, 36.4% moderate and 48.5% severe sleep apnea. A summary of the used part of SHHS-1 dataset is presented in Table 1.

Additionally, for the multiclass classification accuracy assessment, the PhysioNet Sleep Database was used. This database is provided by St. Vincent’s University Hospital and University College Dublin [36,37]. The collection consists of 25 records: 4 of female and 21 of male patients. Disease severity distribution for this dataset is as follows: 4% normal, 40% mild, 24% moderate and 32% severe sleep apnea. All of the records were evaluated. The PhysioNet dataset summary is presented in Table 2.

## 3. Results

The proposed architecture has been validated using the 5-fold cross-validation scheme. For this purpose, the aforementioned subset of records from the SHHS-1 database was used.

For each experiment iteration, these 1000 examinations were divided into the following groups:

- A total of 700 training records;
- A total of 100 validation records;
- A total of 200 test records.

The training subset, was used for training the neural networks, and validation subset for optimizing thresholds of the algorithm. Test records were held out and used only for assessing performance of the method.

The PhysioNet Sleep Database was used only as an additional test set. Neither neural networks nor thresholds were trained on these data. The database was adapted to be processed by the proposed architecture. The approach adopted in this comparison assumed the proposed model to be trained on the SHHS-1 database and validated using the PhysioNet Sleep Database. Trained models were taken from the previous experiment, resulting in five iterations of inference to be conducted. Threshold values used for episode filtering were also reused. The same set of signals from the PhysioNet Sleep Database was processed by all instances of the model.

Signals in the PhysioNet Sleep Database have sampling rate of 8 Hz; these were upsampled to 10 Hz for compatibility with already trained models. For this purpose, the linear interpolation method was used. The airflow sensor used was a thermistor instead of a thermocouple. Episodes labeled as “Unsure” and “Periodic breathing” were excluded from the analysis.

### 3.1. Episode Type Classification Accuracy

One of the most important features of the proposed approach is its ability to discern between the apnea and hypopnea episodes. In order to check the accuracy of individual episode classification, final labels generated by the model were compared to the original annotations from the database for all of the recordings. For every 0.5 s, which is the resolution of the proposed method, predicted class was checked against the true class (either normal, apnea or hypopnea). Then, confusion matrix was constructed containing these microscale results. The above steps have been done for both SHHS-1 test set and PhysioNet Sleep Database.

Table 3 contains a summary of the results. For each fold, percentage of correctly classified samples belonging to each class is presented (i.e., values from the diagonal of their respective confusion matrix). Total accuracy was calculated as a sum of correctly classified samples divided by the total number of samples in the dataset.

**Figure 6:**
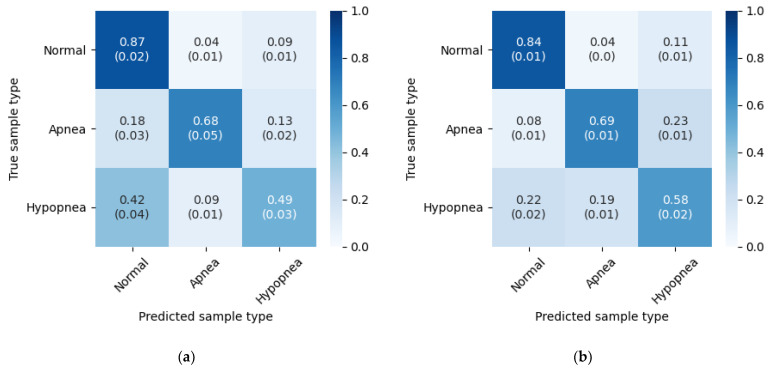
Three class inference confusion matrices, mean and standard deviation from 5 experiment iterations. Values are row-normalized and rounded to two decimal places. (**a**) Test set from SHHS-1 database; (**b**) PhysioNet Sleep Database.

The results from the five experiment iterations were summarized and presented in Figure 6. Mean and standard deviation were calculated for each field.

The values reveal higher accuracy for apnea detection, than for hypopnea. Flow limitation during apnea episodes is more radical, thus they could be easier to detect. The most noticeable difference in the results obtained on different databases is in hypopnea detection accuracy. With the PhysioNet database it is higher by 9% than with the test set from the SHSS-1 database. This can be due to more consistent hypopnea annotations in the PhysioNet Sleep Database.

### 3.2. REI Estimation Quality Evaluation

One of the main purposes of the presented model is to provide an accurate detection of apnea and hypopnea episodes. This can be summarized by REI. To evaluate the performance of the model, in each experiment iteration the REI values have been calculated for 200 test examinations, and then for 25 examinations from the PhysioNet database. Each value is a sum of separately calculated AI and HI indexes, presented for easy comparison with other methods. The examinations were divided into four classes, depending on the REI index. Standard class borders were selected that reflect severity of sleep apnea. They are associated with health effects of the disease, as described in [38]:

- REI < 5—Normal;
- A value of 5 ≤ REI < 15—Mild;
- A value of 15 ≤ REI < 30—Moderate;
- A value of 30 ≤ REI—Severe.

Results obtained with each instance of our model are presented in Table 4. Values represent the percentage of each class that was correctly classified.

**Figure 7:**
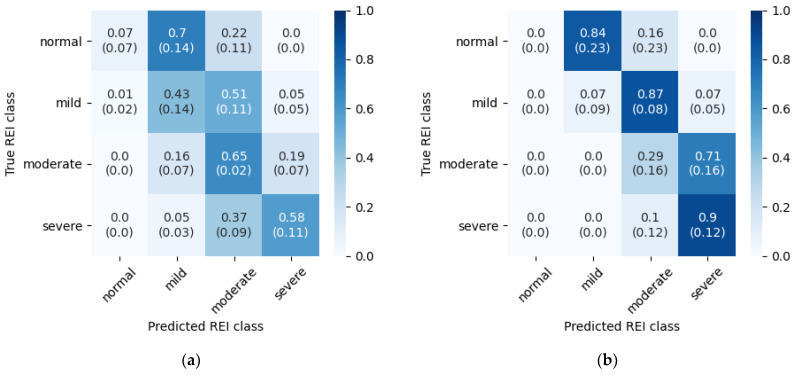
Row-normalized confusion matrices for REI classification (**a**) Test set from SHHS-1 database; (**b**) PhysioNet Sleep Database.

To summarize REI estimation performance, row-normalized confusion matrices have been prepared for both databases (Figure 7). These matrices were obtained by normalization of predictions aggregated from five experiment iterations. Each cell represents a part of the true REI class assigned to the respective predicted REI class, along with standard deviation of these accuracies between folds.

**Figure 8:**
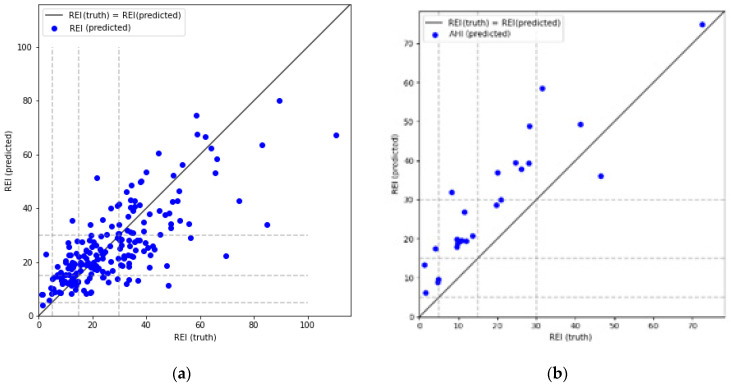
REI estimation graph for one of the experiment iterations (**a**) Test set from SHHS-1 database; (**b**) PhysioNet Sleep Database.

The above results reveal that the model tend to overestimate REI. In the case of SHHS-1 database this is mostly visible in less disordered cases, whereas in the PhysioNet database this behavior spans across the whole dataset. The latter is caused by the threshold determination routine—both models and thresholds were reused from the SHHS-1 experiment, and these thresholds turned out to be too low for the PhysioNet Sleep database. Separate threshold optimization for the PhysioNet database could fix this problem, but with only 25 records available in this dataset it would be difficult to obtain meaningful results. To visualize this behavior, a REI estimation graph from one of the experiment iterations is presented in Figure 8. Dashed lines mark borders between the classes. Many of the misclassified examinations lay close to their target classes. In the case of PhysioNet Sleep Database predictions form a diagonal with a clear positive offset from the correct values.

More detailed results of REI, HI and AI estimation accuracy are provided in Table 5. It can be seen that hypopnea detection is responsible for the majority of REI estimation inaccuracy. This may be due to a fact that hypopnea scoring is normally dependent on desaturation, and this algorithm does not use SpO_2_ signal. The influence of desaturation criteria on the scoring of hypopnea events for SHHS-1 dataset was discussed in [39].

### 3.3. Wake Time Influence on Scoring Performance

**Figure 9:**
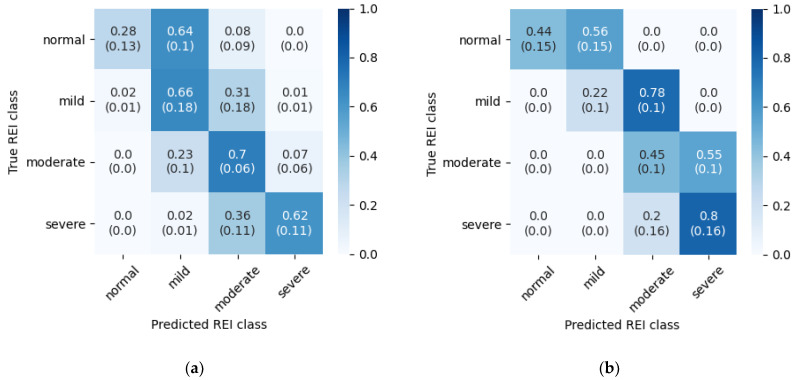
Row-normalized REI classification confusion matrix with “Wake” periods removed. (**a**) Test set from SHHS-1 database; (**b**) PhysioNet Sleep Database.

It has been observed in the SHHS-1 database, that many of the evident breathing events were not annotated. Closer inspection revealed that these episodes frequently lie inside a period of time marked as “Wake”. It seems that there are recordings that lack scoring of respiratory events during wake time. Sleep staging is not a part of this work, nevertheless to estimate the influence of these discrepancies another evaluation was run. For both test datasets (SHHS-1 test set and PhysioNet Sleep Database) parts of the signal marked as “Wake” were not analyzed during this second experiment. All of the episodes during wake time were removed—both in original annotations and in detected epochs. In this way, only disturbances that occurred during actual sleep contribute to the final REI index. Figure 9 contains confusion matrix for disease severity classification, and Table 6 summarizes errors in REI, AI and HI estimation.

In the case of SHHS-1 test dataset, the exclusion of respiratory events detected during wake time resulted in major improvement of REI estimation accuracy, especially in less disordered classes. Such a result could mean that the model is misled by abrupt changes in the airflow that occurred when the patient was not sleeping. The PhysioNet Sleep Database results exhibit similar behavior—REI estimation without wake periods is still shifted towards more diseased cases, but the effect is not as substantial as in the original evaluation. This experiment shows that database quality is very important to accurately assess the performance of such a method.

**Figure 10:**
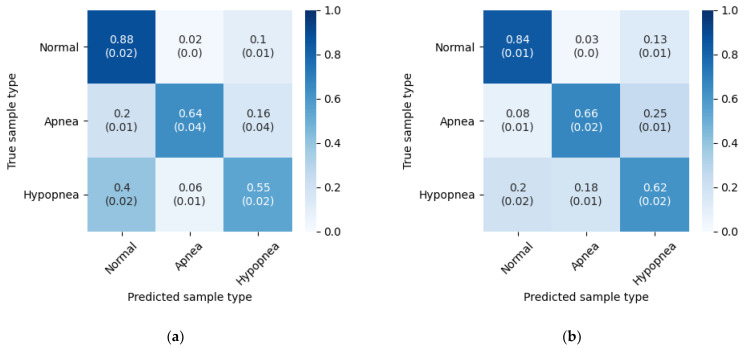
Three-class inference confusion matrix, mean and standard deviation from 5 experiment iterations. “Wake” periods removed. Values are row-normalized and rounded to two decimal places. (**a**) Test set from SHHS-1 database; (**b**) PhysioNet Sleep Database.

Episode classification accuracy was also tested on both databases with wake periods removed. The method is the same as with the original experiment. Table 7 contains accuracies for five experiment iterations, and Figure 10 presents confusion matrices with average accuracies and standard deviations. The results below can be compared directly with Table 3 and Figure 6.

The wake periods removal led to increased hypopnea episodes detection accuracy, but lowered apnea performance slightly. This indicates that the problem inside the “Wake” periods is mainly with hypopneas. An additional analysis of SpO_2_ signal could improve the results, as drops in the blood oxygen saturation may not be so significant when the patient is not sleeping. Additionally, the SHHS-1 database results got improved in the normal breathing class, which is not the case with the PhysioNet Sleep Database. This difference may indicate that the annotations in the latter are more consistent during various sleep stages.

### 3.4. Additional Episode Marking Effect

**Figure 11:**
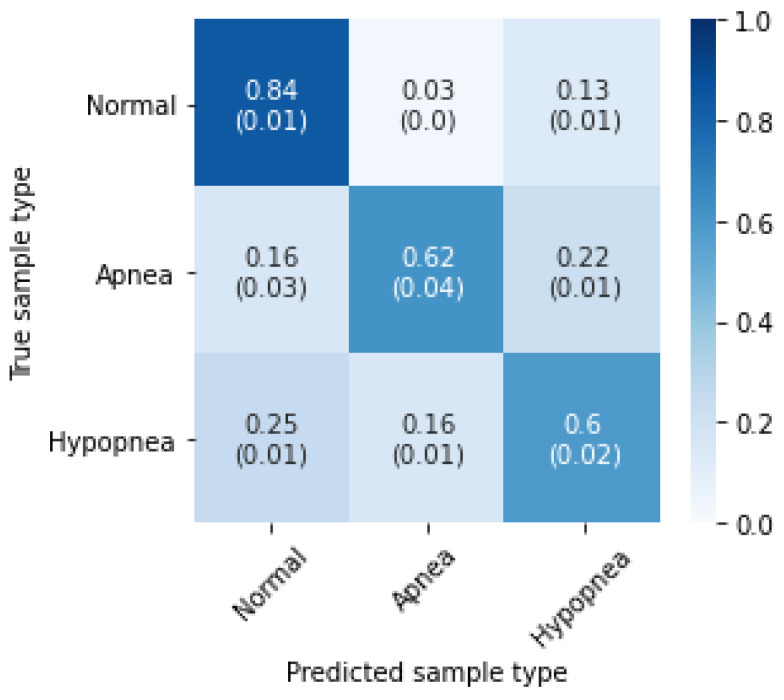
Three-class inference confusion matrix, mean and standard deviation from 5 experiment iterations on PhysioNet Sleep Database, without additional episode marking based on the N class probability. Values are row-normalized and rounded to two decimal places.

Originally, additional episode marking based on the value of the N class was not performed. The apnea and hypopnea episodes were annotated based solely on the values of A and H class probabilities, respectively. However, after close scrutiny we have found out that there are episodes for which the results were ambiguous—the model was not sure to which class of disordered breathing they belong and left them marked as normal breathing. This happened despite the fact that the average N class probability for these events was very low. With this in mind we have added these additional episodes annotation to the postprocessing stage. The confusion matrix representing three-class inference performance on the PhysioNet Sleep Database without additional episode marking is presented in Figure 11.

The most noticeable difference is lower apnea accuracy for the method without additional episodes. This is because in case of many episodes the results were ambiguous and such events were not marked (i.e., they were left marked as normal breathing). For the same reason there are also more false negatives for both apnea and hypopnea events. However, the percentage of hypopnea samples that are correctly classified is lower after annotating the additional episodes, but the difference is only 2%. This is caused by the automatic threshold calculation procedure—both A and H thresholds tend to be higher after the addition of the new events. The addition of this step also helped to stabilize performance between folds slightly.

## 4. Discussion

The main goal of the proposed algorithm is to detect sleep disordered breathing episodes and classify them as either apnea or hypopnea. As was discussed earlier, many publications focus on delivering similar differentiation, but most of them have limitations that render them hard to compare with the proposed model. The algorithm with the closest end goal was described in work [28] and therefore performance of the proposed algorithm was compared to this publication. The other work was developed and tested using PhysioNet Sleep Database. In contrast to [28], where two records were discarded from study due to poor signal quality, in this work all of the examinations available in the database were used. In the above setup the accuracies obtained by our method outperform the values reported in [28] and shown in Table 8. Moreover, our results were obtained using models and thresholds trained only on the SHHS-1 database. This approach has confirmed that the proposed architecture can be portable between databases.

Unfortunately, Ref. [28] does not consider REI estimation accuracy. Therefore, this parameter cannot be compared with CNN from the other publication.

Another study that aimed to differentiate between apnea and hypopnea episodes was described by Nikkonen et al. [40]. It also uses LSTM networks to deliver class probabilities, but there is no postprocessing stage. The other algorithm differs from the one described in that it uses slightly different set of signals—most notably SpO_2_ is required as well as two nasal flow sources, both thermistor and pressure. Only data segments between “lights off” and “lights on” were included in the other study, whereas the presented results were obtained on complete recordings. The other work is based on a newer database that seems to not be publicly available, which is important when comparing the performance of the two approaches. Mean absolute errors of AHI, HI and AI estimation from [40] are presented in Table 9.

The other approach seems to be accurate in episode detection. Apnea and hypopnea detection accuracies averaged across the patients are reported to be 78.3% and 54.7%, respectively. However, the method of obtaining these values is different than in the presented work. In [40], the event is considered correct when it overlaps with an event labeled by a sleep technician, and in this work every 0.5 s of the recording is taken into account separately in accuracy calculation. In the other paper, average episode start and end time errors for apneas were calculated as 7.0 and 1.2, respectively, and for hypopneas they were 6.7 and 2.6, respectively. Given 10s of minimal episode duration these values seem to be significant.

The second approach used to assess the performance of the new method was to check REI estimation accuracy. In the first place, level of agreement could be checked against this of two human experts. The values from Table 5 can be compared with inter-scorer variability. In [41] mean absolute error for AHI calculation was presented for five different scorers. These errors range from 3.82 to 5.15. Our proposed method does not have such a high agreement. This is mainly caused by hypopnea events misclassification, which in turn can originate from the lack of SpO2 signal that is crucial for scoring desaturations needed for accurate hypopnea annotation.

Similar classification task was considered in [15]. Both of the two approaches use the LSTM networks operating on time series data. For this reason, the performance of both algorithms was compared. However, comparison of such results in a form presented in Figure 7. (e) of [15] can be problematic due to different number of examinations used for validation in each work, and changes in the number of records belonging to particular classes between folds. These fluctuations were also reflected in standard deviation provided in Figure 7. (e) of [15].

Figure 7a also contains standard deviation of these accuracies between folds. For the results from [15] standard deviation is not provided, because the other work does not contain results for each fold separately. While summarized confusion matrix is sufficient for calculating per-class normalized accuracy values, results from individual folds should be first normalized to correctly calculate standard deviation as presented in Figure 7a. REI estimation accuracies from [15] are presented in Table 10.

The proposed method exhibits higher accuracy in normal, mild and moderate classes, and lower in severe class than the model from [15]. It is worth noting, that in both approaches there were no cases of severe apnea being misclassified as normal breathing. This fact was highlighted in [15]. Moreover, the presented approach returned no moderate cases as normal, and no normal cases as severe. Another important factor is the difference in how both methods handle wake time during recording. In this paper, total recording time (TRT) was used as denominator for REI calculation, whereas in [15] pure AHI index was used instead. The sleep time is required for AHI calculation, according to AASM rules [1]. Algorithms also differ in that the other approach does not consider classification of detected epochs. Our method uses three respiratory signals instead of just one used in [15]; however, all of these signals are recorded during standard polygraphy anyway.

Both the proposed setup and the one form [15] tend to overestimate apnea severity in less disordered cases. This can be caused by uneven distribution of disease severity across the SHHS-1 dataset used for training of both models. Normal and mild cases make up only 15.1% of the whole dataset. The authors of the other work hypothesize that normal breathing dynamics change with disease severity. The results reflect these differences, because such a model is not able to sufficiently learn the signal characteristics of healthy patients. This problem could potentially be fixed by changing the structure of the used dataset. Methods that do not directly use raw respiratory signals could be more immune to such an imbalance, because they do not rely on breathing dynamics as much.

As AHI estimation is one of the most important tasks performed by this algorithm, its performance was also compared to other methods that were validated on the same database.

Table 10 presents row-normalized results from [25] (Figure 7, SHHS1). The other method compares favorably to the proposed method in terms of the disease severity estimation. However, the method from [25] contains a linear regression stage at the end of the processing pipeline that inflate AHI predictions; therefore, the performance obtained by simply counting detected episodes would be different. Additionally, no apnea/hypopnea classification was done by the other method, as only one class of disordered breathing was considered.

Due to very similar approach, the results of the proposed technique were compared to those of the LSTM from [40]. The other work reports higher accuracies in all classes; however, they were not obtained on the same database.

Row-normalized AHI severity estimation results from [26] are presented in Table 11. Class definitions used in the other work had lower granularity, hence the output of our algorithm was recalculated to match them. The accuracy of the proposed model is worse by a few percent in each class. However, as stated by the authors, their algorithm was neither meant to provide exact locations of the events, nor classify them as apnea or hypopnea type.

The recent work that accomplished most similar task to the presented one on the SHHS-1 database was described in [27]. The other algorithm was able to determine the exact location and duration of apnea and hypopnea episodes, as well as differentiate one from the other. In this regard the goals of [27] and this work were the same. Unfortunately, results for individual episode classification accuracy were not provided, thus the two models cannot be compared in this regard. The difference between the two works is in the kind of algorithm; Ref. [27] does not use a deep learning approach and instead focuses on extensive analysis of the signal itself. Additionally, the SpO_2_ signal is required by the other model. Table 12 shows results from [27] and this work in the form of diagonal values from row-normalized confusion matrices for three AHI cut-offs: 5, 15 and 30.

## 5. Conclusions

Sleep apnea is a relatively common disorder, and public awareness of its consequences is rising [42]. This increases the demand for quick diagnosis. One way of improving efficiency of testing is to provide an aid for technicians that would allow them to shorten the time needed for analyzing a single examination. Automatic sleep disorder detection algorithms are capable of providing initial labels for the events in the signal. This description can be further adjusted by a technician to correct mistakes that may happen.

The aim of this work was to develop a machine learning architecture, capable of providing both an accurate sleep disordered breathing episode detection and good REI estimation. Special and novel feature of the designed model is its capability to distinguish between apnea and hypopnea events. Most publications focus on one of these tasks, either being it REI estimation or apnea/hypopnea differentiation.

Presented approach utilizes novel architecture of two LSTM neural networks arranged in a series, along with signal preprocessing stage and result filtering. Additionally, few additional novel techniques, allowing for better episode detection and classification, have been proposed. The most important are as follows:

- Respiratory signals phase alignment;
- Two-stage architecture with the first stage detecting apnea and beginning of hypopnea;
- Classification thresholding method based on average score of labels in the episode.

Model training and verification of REI estimation accuracy were done on a subset of records from the SHHS-1 database, and additional verification was run on the PhysioNet Sleep Database. The 5-fold cross-validation scheme was used to obtain five independent models. The quality of disease severity class prediction based on REI estimation was compared with several other models that were validated on the same SHHS-1 database. Our proposed model showed slightly worse performance for this task than other methods—however, these methods do not differentiate between the types of the episodes, or do not provide results for quality of such differentiation.

REI estimation quality was also assessed using PhysioNet Sleep Database. The results show overestimation of the predictions on this dataset. This is most probably caused by the automatic threshold determination routine, which is run on the SHHS-1 dataset only. Thresholds obtained seem to be too low for the other database, as there is a constant offset of predictions from the correct values. This is a potential place for future improvement of the algorithm, to be better scalable between databases.

Performance of the model was also assessed on both databases rectified by excluding the so called “wake” periods. This experiment resulted in increased REI estimation accuracy, especially for less disordered cases. Hypopnea detection performance was also better in this case, and apnea annotation quality slightly worse. It can be hypothesized that the results of the method would improve when more accurate database would be used, as many of the respiratory events in periods marked as “Wake” are not accurately annotated in the SHHS-1 database.

These experiments have shown, that the proposed algorithm gives good results, but there are other methods with particularly better AHI severity classification capability. However, with the apnea and hypopnea episodes divided into separate classes by our automatic approach, sleep technicians can immediately have better insight into the examinations. The main advantage of the proposed architecture is that all of the tasks are reliably done by a single model.

The presented method itself has also some drawbacks. Three signals must be available to perform inference with the designed model. The recordings must be of good quality to give proper results; however, this can be simply checked by calculating the energy of the signals. Furthermore, a large part of the available data is discarded during training. While this is not a problem when using big database such as SHHS-1, the model could be hard to train on small databases like the PhysioNet Sleep Database. Additionally, while the model differentiates between apnea and hypopnea episodes, it does not mark the type of an epoch, i.e., if it was central, obstructive or mixed. Separating obstructive and central episodes can be considered as an addition to the presented method—another model could be easily designed as a next stage for providing this additional classification which is a promising area for future development. The architecture also needs certain processing power to be trained efficiently. Another matter worth noting is that the presented approach does not use SpO_2_ signal. It is a possibility for future development to integrate blood oxygen saturation analysis into this model to achieve better scoring accuracy. However, in the present form the system is able to annotate the examinations even when the SpO_2_ signal is not available due to sensor malfunction or disconnection. These factors, however, do not limit the possibility of using the proposed solution as an initial scoring algorithm for at-home sleep studies.

The advantage and the main use case of the proposed method is to provide apnea and hypopnea labels suggestions for medical personnel. These labels could streamline further human work, as majority of the episodes would be already marked. Therefore, this approach is not meant to be a standalone solution for sleep study scoring, rather it is a tool to speed up the whole process.

## Figures and Tables

**Table 1:** Summary of the used part of SHHS-1 dataset.

Parameter	Mean	Standard Deviation
Age (years)	58.24	11.94
Weight (kg)	79.19	16.45
BMI (kg/m^2^)	27.57	5.12
Recording duration (h)	7.28	0.97
AHI	33.33	19.34

**Table 2:** Summary of the PhysioNet dataset.

Parameter	Mean	Standard Deviation
Age (years)	49.96	9.35
Weight (kg)	95.02	14.41
BMI (kg/m^2^)	31.6	3.95
Recording duration (h)	6.93	0.53
AHI	24.1	20.3

**Table 3:** Three-class inference performance of each instance of the model. Values for SHHS-1/Physionet datasets.

Average Accuracy (%)	Fold 1	Fold 2	Fold 3	Fold 4	Fold 5
Normal breathing	86.56/84.48	87.07/85.30	88.06/85.10	87.81/84.42	82.61/82.47
Hypopnea	47.19/54.69	48.21/56.41	46.36/59.18	50.54/60.80	54.20/60.32
Apnea	69.60/70.34	60.61/70.97	68.69/70.23	66.29/67.34	75.83/68.67
Total	80.52/81.90	80.59/82.78	81.65/82.80	82.25/82.24	78.31/80.48

**Table 4:** Apnea severity estimation accuracy of each instance of the model. Values represent true positives for each class in SHHS-1/PhysioNet databases.

Sleep Apnea Severity Class	Fold 1	Fold 2	Fold 3	Fold 4	Fold 5
Normal	14%/0%	0%/0%	0%/0%	14%/0%	0%/0%
Mild	52%/11%	41%/22%	56%0%	47%/0%	16%/0%
Moderate	61%/43%	68%/43%	63%/29%	66%/29%	66%/0%
Severe	53%/75%	48%/75%	51%100%	57%/100%	79%/100%

**Table 5:** Mean error, standard deviation, min and max errors, Pearson’s and Spearman’s correlation coefficients for REI, HI and AI. The values were averaged across five experiment iterations. Data obtained on SHHS-1/PhysioNet databases.

	Mean Absolute Error	Standard Deviation	Min Error	Max Error	Pearson’s r	Spearman’s r
REI	9.24/10.52	11.61/7.92	−27.07/−27.52	46.58/14.67	0.71/0.88	0.67/0.89
HI	8.99/9.16	10.76/8.36	−21.44/−20.04	47.22/19.36	0.59/0.69	0.57/0.72
AI	3.11/3.28	3.57/4.62	−20.54/−21.55	11.25/2.59	0.86/0.96	0.64/0.65

**Table 6:** Mean error, standard deviation, min and max errors, Pearson’s and Spearman’s correlation coefficients for REI, HI and AI with “Wake” periods removed. The values were averaged across five experiment iterations. Data obtained on SHHS-1/PhysioNet databases.

	Mean Absolute Error	Standard Deviation	Min Error	Max Error	Pearson’s r	Spearman’s r
REI	5.57/7.39	7.55/6.56	−18.13/−17.02	38.58/16.55	0.82/0.88	0.8/0.93
HI	5.61/7.43	7.22/6.95	−16.95/−14.45	33.97/15.85	0.75/0.74	0.73/0.71
AI	1.39/1.57	2.34/3.03	−13.57/−13.35	13.09/3.39	0.91/0.96	0.71/0.76

**Table 7:** Three-class inference performance of each instance of the model with “Wake” periods removed. Values for SHHS-1/Physionet datasets.

Average Accuracy (%)	Fold 1	Fold 2	Fold 3	Fold 4	Fold 5
Normal breathing	88.93/84.76	87.85/83.84	88.86/84.52	89.54/84.74	84.39/81.57
Hypopnea	53.11/57.97	55.36/61.51	54.26/63.43	53.42/63.24	57.55/63.93
Apnea	65.64/66.33	56.63/68.22	62.68/68.55	66.47/63.42	67.37/66.27
Total	82.41/81.78	81.52/81.34	82.59/82.13	83.37/82.18	79.13/79.52

**Table 8:** Three-class inference accuracy of the CNN from [28].

Average Accuracy	CNN from [28]
Normal breathing	82.20%
Hypopnea	53.61%
Apnea	66.24%
Total	79.61%

**Table 9:** Mean absolute error for REI, HI and AI estimation from [40].

Mean Absolute Error	LSTM from [40]
REI	3.0
HI	2.9
AI	2.0

**Table 10:** REI estimation performance in comparison with LSTM from [15], Random Forest from [25] and LSTM from [40].

Average Accuracy (%)	Our work	LSTM from [15]	Random Forest from [25]	LSTM from [40]
REI < 5	7%	3%	51%	92%
5 ≤ REI < 15	43%	26%	67%	81%
15 ≤ REI < 30	65%	54%	74%	82%
30 ≤ REI	58%	73%	86%	92%

**Table 11:** REI estimation performance in comparison with GRU from [26].

Average Accuracy (%)	Our Work	GRU from [26]
REI < 5	7%	32%
5 ≤ REI < 30	86%	94%
30 ≤ REI	58%	69%

**Table 12:** REI estimation performance in comparison with a method from [27] for different cut-offs.

Average Accuracy (%)	Cut-Off = 5		Cut-Off = 15		Cut-Off = 30	
	Our Work	[27]	Our Work	[27]	Our Work	[27]
Negative	7%	60%	48%	88%	87%	98%
Positive	100%	99%	90%	95%	58%	88%
